# Impact of critical illness on continuation of anticancer treatment and prognosis of patients with aggressive hematological malignancies

**DOI:** 10.1186/s13613-024-01372-5

**Published:** 2024-09-11

**Authors:** Swann Bredin, Justine Decroocq, Clément Devautour, Julien Charpentier, Clara Vigneron, Frédéric Pène

**Affiliations:** 1 Service de médecine intensive-réanimation, hôpital Cochin, Assistance Publique-Hôpitaux de Paris. Centre, Université Paris-Cité, Paris, France; 2 Service d’hématologie clinique, hôpital Cochin, Assistance Publique-Hôpitaux de Paris. Centre, Université Paris-Cité, Paris, France; 3 Institut Cochin, INSERM U1016, CNRS UMR8104, Université Paris-Cité, Paris, France

**Keywords:** Leukemia, Lymphoma, Chemotherapy, Outcome, ICU

## Abstract

**Background:**

Maintaining the dose-intensity of cancer treatment is an important prognostic factor of aggressive hematological malignancies. The objective of this study was to assess the long-term outcomes of intensive care unit (ICU) survivors with acute myeloid leukemia (AML) or aggressive B-cell non-Hodgkin lymphoma (B-NHL) with emphasis on the resumption of the intended optimal regimen of cancer treatment.

**Patients and methods:**

We conducted a retrospective (2013–2021) single-center observational study where we included patients with AML and B-NHL discharged alive from the ICU after an unplanned admission. The primary endpoint was the change in the intended optimal cancer treatment following ICU discharge. Secondary endpoints were 1-year progression-free survival and overall survival rates. Determinants associated with modifications in cancer treatment were assessed through multivariate logistic regression.

**Results:**

Over the study period, 366 patients with AML or B-NHL were admitted to the ICU, of whom 170 survivors with AML (*n* = 92) and B-NHL (*n* = 78) formed the cohort of interest. The hematological malignancy was recently diagnosed in 68% of patients. The admission Sequential Organ Failure Assessment (SOFA) score was 5 (interquartile range 4–8). During the ICU stay, 30 patients (17.6%) required invasive mechanical ventilation, 29 (17.0%) vasopressor support, and 16 (9.4%) renal replacement therapy. The one-year survival rate following ICU discharge was 59.5%. Further modifications in hematologic treatment regimens were required in 72 patients (42%). In multivariate analysis, age > 65 years (odds ratio (OR) 3.54 [95%-confidence interval 1.67–7.50], *p* < 0.001), ICU-discharge hyperbilirubinemia > 20 µmol/L (OR 3.01 [1.10–8.15], *p* = 0.031), and therapeutic limitations (OR 16.5 [1.83–149.7], *p* = 0.012) were independently associated with modifications in cancer treatment. Post-ICU modifications of cancer treatment had significant impact on in-hospital, 1-year overall survival and progression-free survival.

**Conclusion:**

The intended cancer treatment could be resumed in 58% of ICU survivors with aggressive hematological malignancies. At the time of ICU discharge, advanced age, persistent liver dysfunction and decisions to limit further life-support therapies were independent determinants of cancer treatment modifications. These modifications were associated with worsened one-year outcomes.

**Supplementary Information:**

The online version contains supplementary material available at 10.1186/s13613-024-01372-5.

## Introduction

The incidence of aggressive hematologic malignancies, such as acute leukemias and aggressive lymphomas, is continuously increasing within the general population [1]. In relation with the underlying disease or with its treatment, patients harbor a high risk for life-threatening complications that warrant ICU admission. A statewide study in Canada thus reported that the considerable proportions of 22% and 17% of patients with acute leukemia or aggressive lymphoma required ICU admission within the first year following diagnosis [2]. Acute leukemia and aggressive lymphoma thereby account for the majority of hematological malignancies encountered in the ICU. The use of critical care has been modified towards earlier ICU admission policies, along with less invasive diagnostic and therapeutic strategies [3–6]. Most importantly, ICU survival has considerably improved over time, at the expense of disability, impaired functional and cognitive status and persistent organ dysfunctions after ICU discharge [7–10]. Beyond short-term survival outcomes, the improved prognosis of critical care in patients with hematological malignancies now raises the question on long-term outcomes, in terms of survival as well as achievement or maintenance of sustained remission.

The prognosis of aggressive hematological malignancies relies on intrinsic tumor-related factors, but also on patients’ characteristics and their eventual capacities to sustain the desired dose-intensity of chemotherapy in narrow therapeutic windows [11, 12]. The consequences of ICU-acquired frailty on the continuation of specific hematological treatment and the long-term hematological and vital prognosis has thus become a relevant field of investigation [13–16]. The goal of this study was to assess the long-term outcomes of ICU survivors with acute myeloid leukemia (AML) or aggressive B-cell non-Hodgkin lymphoma (B-cell NHL), with emphasis on the continuation of the intended optimal cancer treatment regimens.

## Patients and methods

### Patients

We conducted a retrospective 9-year (2013–2021) single-center study in a 24-bed medical ICU of a tertiary care hospital with a dedicated hematology unit. We included patients with AML or aggressive B-cell NHL followed up in our hospital, who had required unplanned ICU admission and had been eventually discharged alive from the ICU. Autologous and allogeneic hematopoietic stem cell transplant recipients were excluded, as well as patients in complete remission without ongoing treatment or patients in palliative care prior to ICU admission and not eligible to further cancer treatment. For patients with multiple ICU admissions during the study period, only the first admission was considered. Besides, a local control cohort of consecutive patients with AML or aggressive B-cell NHL who had not required ICU admission during the period 2017–2023 was identified through the AP-HP clinical database warehouse, without additional matching rule. The study was conducted following the principles of the Helsinki Declaration and was approved by the ethics committee of the French Intensive Care Society (CE SRLF 24 − 023, IRB # 00014135). The retrospective and observational nature of the study waived the need for patient consent.

### Data collection

We collected the main features of the underlying malignancy, including cytological or histological subtype and disease status. AML were classified into three prognostic groups (favorable, intermediate, and poor) based on cytogenetics and molecular characterization according to European LeukemiaNet 2017 classification [17]. Stage of disease for B-cell NHL was reported according to the Ann Arbor classification, the International Prognostic Index score and the Burkitt Lymphoma-modified International Prognostic Index score as appropriate [18, 19]. The intended optimal therapeutic regimen was established by the referring haematologist based on the concurrent guidelines. The ICU stay was characterized by the following variables: reason for admission, severity scores Sequential Organ Failure Assessment (SOFA) and Simplified Acute Physiology Score 2 (SAPS2), extent of organ supports, decisions to limit life-sustaining therapies, in-ICU length of stay [20, 21]. Therapeutic limitations on further readmission to the ICU or initiation of invasive life supports were discussed in multidisciplinary rounds and implemented at the time of ICU discharge. Acute kidney injury was defined as stage 1 or more of the KDIGO classification [22]. After ICU discharge, all patients were followed-up for one year. It included vital status as well as hematological outcomes, including remission, progression and relapse, as determined by the attending hematologist [23, 24]. We recorded the characteristics of cancer treatment after the ICU stay (drug regimens and doses) and reasons for eventual changes in the intended therapeutic regimen.

### Outcomes

The primary endpoint was the change in the intended optimal cancer treatment following ICU discharge. Secondary endpoints were 1-year progression-free survival and overall survival rates.

### Statistical analysis

Descriptive statistics are presented as percentages for categorical variables, or median and interquartile range for continuous variables. Categorical and continuous variables were compared using the chi-square test and the Mann-Whitney U test, respectively. Variables associated with modification of treatment in the univariate analysis at a *p* = 0.10 level were entered into a multivariate logistic regression model, with results presented as odds ratios and their 95% confidence intervals. Progression-free survival and overall survival curves were built using Kaplan-Meier method and compared by log-rank test. Statistical analysis was carried out using SPSS 25.0 (SPSS Inc, Chicago, Illinois, USA) and R Statistical Software (v4.3.2; R Core Team 2023).

## Results

**Fig. 1:**
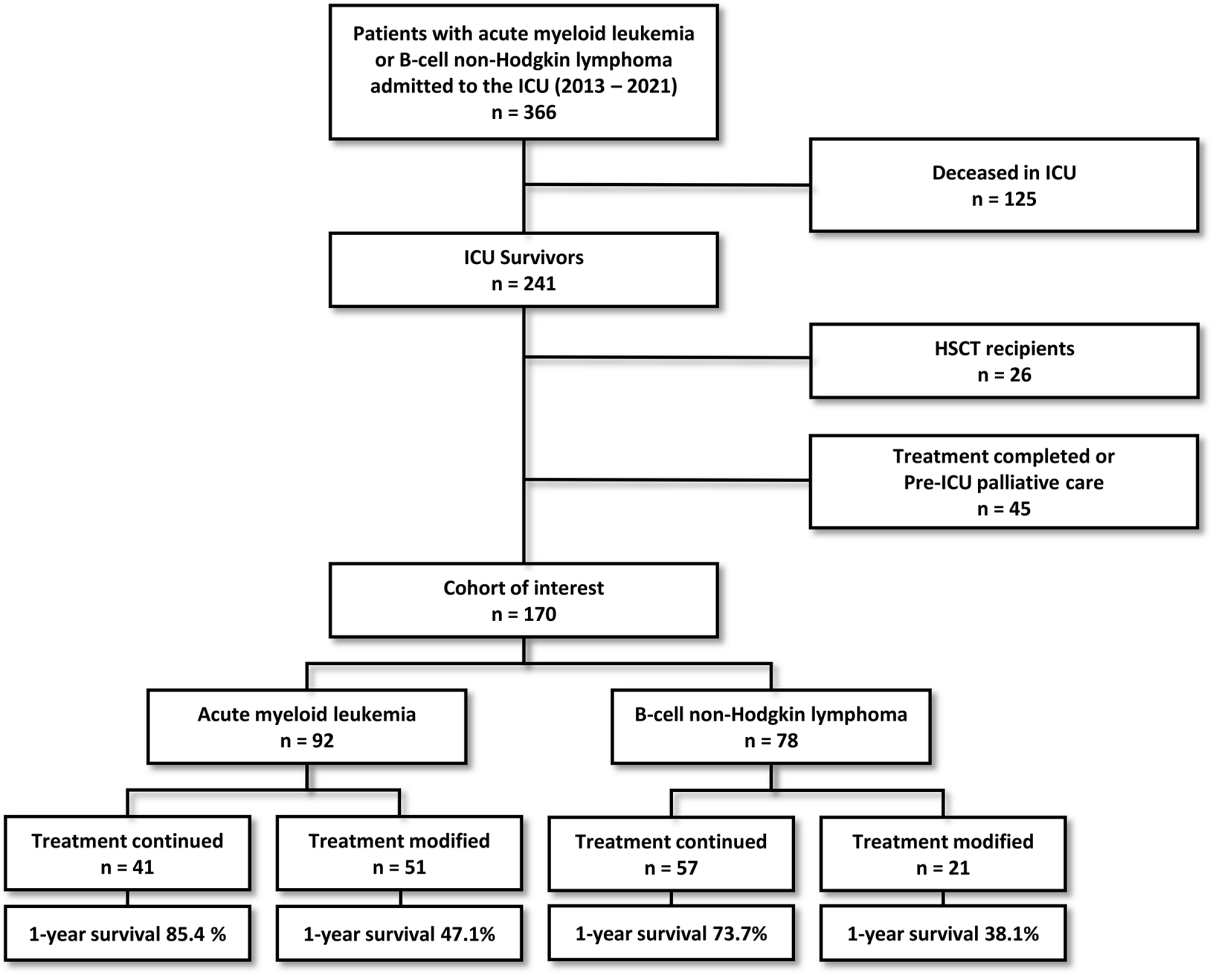
Flow chart of the study. AML: acute myeloid leukemia, B-NHL: B-cell non-Hodgkin lymphoma, HSCT: hematopoietic stem cell transplant

During the 9-year study period, 366 patients with AML or B-cell NHL were admitted to the ICU. Among them, 170 ICU survivors with AML (*n* = 92) and B-cell NHL (*n* = 78) formed the cohort of interest (Fig. 1). Their characteristics are displayed on Tables 1 and 2. The median age of the patients was 61 [48–72] years, and 55.2% were males. The 92 AML cases were distributed into three prognostic subgroups groups (16.3% favorable, 42.3% intermediate, 41.3% poor). B-cell NHL were mostly diffuse large B-cell lymphoma (74.3%) and Burkitt lymphoma (12.3%) and almost all of them displayed advanced stages III/IV. A large majority of patients (68%) had recent diagnosis made within the month prior to ICU admission or during the ICU stay. The median admission SOFA score and SAPS2 were 5 [4–8] and 42 [31–51] points, respectively. During the ICU stay, 30 patients (17.6%) required invasive mechanical ventilation, 29 (17.0%) vasopressor support, and 16 (9.4%) renal replacement therapy. Two patients remained dependent of haemodialysis after ICU discharge. The median in-ICU length of stay was 3 days [2–6]. Twelve patients were subjected to therapeutic limitations towards further ICU re-admission (supplementary Table 1). The overall 6-month and one-year survival rates following ICU discharge were 71.2% and 59.5%, respectively.

**Table 1:** Characteristics of patients according to further continuation of hematological treatment IQR : interquartile range, DLBCL : diffuse large B-cell lymphoma, IPI : international prognostic index, BL-IPI, Burkitt’s lymphoma international prognostic index

Variables, *n* (%) or median [IQR]	All ICU survivors (*n* = 170)	Treatment continued (*n* = 98)	Treatment modified (*n* = 72)	*p*
Demographics				
Age (years)	61 [48–72]	59 [42–67]	69 [54–75]	0.004
Male gender	94 (55.2)	59 (60.2)	35 (52.2)	0.295
Comorbidities				
Hypertension	40 (23.5)	20 (20.4)	20 (29.8)	0.077
Diabetes mellitus	17 (10.0)	10 (10.2)	5 (7.4)	0.941
Chronic kidney disease	6 (3.5)	3 (3.0)	3 (4.4)	0.697
Chronic lung disease	3 (1.7)	3 (3.0)	0	0.265
Chronic heart failure	3 (1.7)	2 (2.0)	1 (1.4)	0.455
Hematological malignancy				
Acute myeloid leukemia	92 (54.1)	41 (41.8)	51 (70.8)	< 0.001
Cytogenetic risk				< 0.001
Favorable	15 (8.8)	13 (13.2)	2 (2.7)	
Intermediate	38 (22.3)	13 (13.2)	25 (34.7)	
Adverse	39 (22.9)	15 (15.3)	24 (22.3)	
DLBCL	59 (34.7)	40 (40.8)	19 (26.3)	0.032
Stage III/IV	57 (33.5)	39 (39.7)	18 (25.0)	0.008
IPI score > 2	41 (24.1)	24 (24.7)	16 (22.2)	0.175
Burkitt lymphoma	10 (5.8)	9 (9.1)	1 (1.5)	0.046
BL-IPI ≥ 2	6 (3.5)	5 (5.1)	1 (1.3)	0.397
Primary cerebral lymphoma	10 (5.8)	8 (8.1)	2 (3.03)	0.193
Disease status				0.795
Inaugural	116 (68.2)	68 (69.3)	48 (66.6)	
Relapse or progression	34 (20.0)	19 (19.3)	15 (20.8)	
Remission	20 (11.8)	11 (11.2)	9 (12.5)	

**Table 2:** ICU characteristics and survival outcomes of patients according to further continuation of haematological treatment IQR : interquartile range, ICU : intensive care unit. SOFA score : sepsis-related organ failure score. SAPS2 : simplified Acute Physiology score 2

Variables, *n* (%) or median [IQR]	All (*n* = 170)	Treatment continued (*n* = 98)	Treatment modified (*n* = 72)	*p*
Reason for ICU admission				0.878
Acute respiratory failure	33 (19.4)	18 (18.3)	15 (20.8)	
Circulatory failure	49 (28.8)	26 (26.5)	23 (31.9)	
Metabolic disturbances	23 (13.5)	13 (13.2)	10 (13.8)	
Neurologic disorder	12 (7.0)	8 (8.1)	4 (5.5)	
Septic shock	15 (8.8)	9 (9.1)	6 (8.3)	
Monitoring	37 (21.7)	24 (24.4)	13 (18.0)	
Admission severity scores				
SOFA score	5 [4–8]	4 [3–7]	5 [4–9]	0.009
SAPS2	42 [31–51]	39 [28–51]	45 [36–53]	0.010
In-ICU management				
Invasive mechanical ventilation	30 (17.6)	17 (17.3)	13 (19.4)	0.872
Vasopressors	29 (17.0)	12 (12.2)	17 (25.3)	0.016
Renal replacement therapy	16 (9.4)	10 (10.2)	6 (8.9)	0.941
Chemotherapy	105 (61.7)	53 (54.0)	52 (72.2)	0.016
Characteristics at ICU discharge				
Bilirubinemia > 20 µmol/L	29 (17.0)	10 (10.2)	19 (26.3)	0.006
Bilirubinemia (µmol/L)	10 [6–17]	8 [6–15]	12 [7–21]	0.001
Albuminemia < 25 g/L	45 (26.4)	20 (20.4)	25 (34.7)	0.037
Oxygen requirement	38 (22.3)	17 (17.3)	21 (29.1)	0.09
Acute kidney injury	33 (19.4)	21 (21.4)	12 (16.6)	0.55
Creatininemia (µmol/L)	65 [50–88]	65 [48–88]	66 [52–92]	0.229
Performans status (3–4)	36 (21.1)	18 (18.3)	18 (25.0)	0.344
Therapeutic limitations	12 (7.0)	1 (1.0)	11 (15.2)	0.001
In-ICU length of stay (days)	3 [2–6]	3 [2–5]	3 [2–6]	0.677
Post-ICU outcomes				
Hospital survival	147 (86.5)	94 (96.0)	53 (71.7)	< 0.001
6-month survival	121 (71.2)	82 (83.7)	39 (50.8)	< 0.001
1-year survival	101 (59.5)	71 (72.5)	30 (37.4)	< 0.001

**Fig. 2:**
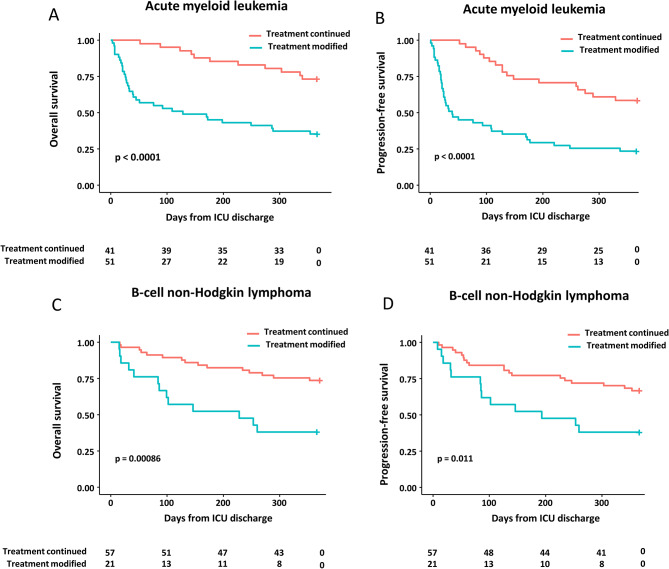
Overall survival and progression-free survival in ICU survivors with acute myeloid leukemia (**A**, **B**) or B-cell non-Hodgkin lymphoma (**C**, **D**) depending on further continuation of hematological treatment. The landmark was set at the time of ICU discharge. P values are obtained from log-rank tests

Following ICU discharge, 72 patients (42.3%) required some modifications of hematologic treatment regimens, including full discontinuation in 39 patients (22.9%, including 30 patients with AML and 9 patients with B-cell NHL), drug changes in 15 patients (8.8%), delayed administration in 11 patients (6.4%), and dose reduction in 7 patients (4.1%). Recorded reasons for treatment modifications were persistent organ failures (31.9%), impaired functional status (34.7%), persistent infectious processes (9.7%), and progression of disease (23.6%). Patients from the “modified treatment” group were significantly older, had higher SOFA and SAPS2 scores at ICU admission, and had been more frequently exposed to vasopressors. Of note, disease status at the time of ICU admission was balanced between the two groups, and in-ICU lengths of stay were similar. At ICU discharge, patients who required treatment modifications exhibited lower albumin and higher bilirubin levels, and were more frequently subjected to decisions for limiting life-sustaining therapies. In multivariate analysis, age > 65 years (odds ratio (OR) 3.54 [95%-confidence interval 1.67–7.50], *p* < 0.001), ICU-discharge hyperbilirubinemia > 20 µmol/L (OR 3.01 [1.10–8.15], *p* = 0.031), and therapeutic limitations (OR 16.5 [1.83–149.7], *p* = 0.012) remained independently associated with further modifications in cancer treatment (Table 3). Post-ICU modifications of cancer treatment had significant impact on in-hospital, 6-month and 1-year overall survival and progression-free survival (Fig. 2). The one-year survival rate was lower in patients with full treatment discontinuation (27.3%) than if partial modifications (59.0%).

**Table 3:** Predictors of modifications in hematological treatment among the 170 ICU survivors

	Univariate			Multivariate		
Variable	**Odds ratio**	**95% CI**	***p***	**Odds ratio**	**95% CI**	***p***
Age > 65 years-old	2.88	1.53–5.42	< 0.001	3.03	1.49–6.16	0.002
Characteristics of ICU stay						
Admission SOFA score	1.12	1.02–1.22	0.011	1.02	0.91–1.15	0.650
Invasive mechanical ventilation	1.15	0.52–2.51	0.726			
Vasopressors	2.75	1.24–6.09	0.012	1.93	0.65–5.66	0.231
Characteristics at ICU discharge						
Performans status (3–4)	1.48	0.70–3.10	0.297			
Hypoalbuminemia (< 25 g/L)	2.07	1.04–4.13	0.038	1.83	0.82–4.05	0.137
Bilirubinemia > 20 µmol/L	3.15	1.36–7.29	0.007	3.35	1.26–8.92	0.015
Acute kidney injury	0.73	0.33–1.60	0.439			
Therapeutic limitations	17.4	2.2-138.9	0.007	16.5	1.83–149.7	0.012

95% CI: 95% confidence interval. ICU : intensive care unit. SOFA : sepsis-related organ failure assessment

**Fig. 3:**
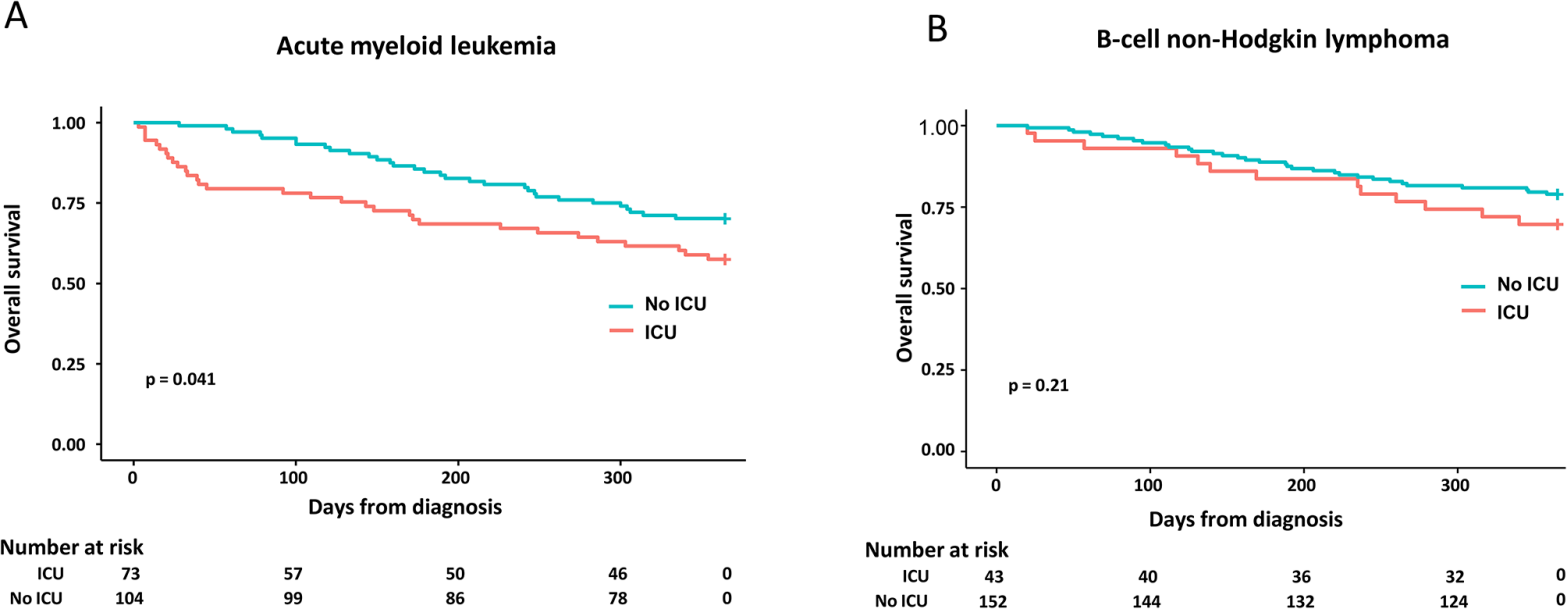
Overall survival in patients with acute myeloid leukemia **(A)** and B-cell non-Hodgkin lymphoma **(B)** admitted or not to the ICU within the first month of diagnosis

Finally, one-year overall survival was compared between patients who survived an ICU stay in the first month of diagnosis and patients who did not require ICU admission (supplementary Tables 2 and 3). One-year survival was significantly impaired in post-ICU AML patients (Fig. 3A), and was similar in post-ICU and non-ICU B-cell NHL patients (Fig. 3B).

## Discussion

The improved prognosis of critically ill cancer patients now imposes to address long-term outcomes beyond the limited short-term objective of ICU and hospital discharge. Transfer to the ICU, once commonly perceived as a terminal event in the course of hematological malignancies, may actually allow a bridge-to-cure in an important proportion of patients. Hence, the results presented here appear very encouraging since the majority of patients were able to resume the intended cancer treatment after the ICU stay, with a dramatic impact on disease control and survival.

In the light of improved survival to critical illnesses, prognostic considerations extend to long-term outcomes, and how acute conditions may jeopardize the course and management of the underlying disease. In a French pivotal study of 1011 patients with hematological malignancies, where AML and NHL accounted for 27.2% and 31.6% of underlying neoplasms, the in-ICU and one year survival rates reached 72.4% and 43.3%, respectively. Of note, almost all ICU survivors had been able to resume their cancer treatment with little influence of the ICU admission on therapeutic intensity, and 80% of ICU survivors were therefore in complete or partial remission at 6 months [25]. Specific cohorts of patients with AML (newly diagnosed in 70% of cases) and newly-diagnosed lymphoma reported consistent one-year survival rates of 45.3% and 49% [26, 27]. Although the short-term outcomes of critically ill patients with malignancies mostly rely upon the extent of organ failures, it has been repeatedly demonstrated that the long-term outcomes of ICU survivors is driven by the particular prognostic determinants of the underlying disease, though the impact on the continuation of treatment had not been extensively addressed. Our results are in line with those from several cohorts of cancer patients with both hematological malignancies and solid tumors suggesting that (i) The long-term prognosis of ICU survivors is somewhat comparable to counterpart patients who never required ICU admission; (ii) the intended cancer treatment could be resumed in most patients. In a pioneer study, Schellongowski and colleagues thus reported comparable six-year survival and progression-free survival rates in ICU and non-ICU patients with *de novo* AML who survived the first 30 days after diagnosis [28]. A recent multicenter Canadian study gathered a cohort of 203 ICU survivors with hematological malignancies, including half of patients with AML or NHL. The overall 12-month survival rate was 40% (28% for AML and 36% for NHL), in relation with severe-to-moderate frailty and impaired functional status at ICU admission. In a subgroup analysis of patients with treatment status available at 6 months (mostly AML and NHL), the intended treatment was continued in 25% and modified in 23%, while 39% were no longer candidates for treatment. Several factors certainly accounted for such discrepancies between that cohort and the present one, including the variable categorization of diseases, depending or not of receipt of hematopoietic stem cell transplantation, and different ICU admission policies. Indeed, a large majority of patients from our cohort were admitted with *de novo* AML or B-cell NHL with inaugural complications [29].

Besides the intrinsic characteristics of the underlying malignancy, the continuation of cancer treatment is emerging as a critical endpoint following recovery from critical illness. This appears particularly relevant to aggressive hematological malignancies where the maintenance of dose-intensity is critical for swift and sustained disease control. Along this line, the present study provides simple criteria to predict the continuation of treatment. However it is noteworthy that most of them may become available only at the time of ICU discharge. Interestingly, neither the severity at admission nor the extent of organ supports during the ICU stay impacted on the continuation of anticancer treatment, which relied on age, therapeutic limitations, as well as persistent liver dysfunction at the time of ICU discharge. Age is often a prominent prognostic factor in acute life-threatening condition since elderly patients harbour a higher prevalence of comorbid conditions, and are prone to various post-ICU complications. Malnutrition, delirium or neuromyopathy and prolonged impaired functional status could not be accurately captured by our study, but may have contributed to the decision-making process of therapeutic limitations [30, 31]. The association of therapeutic limitations with treatment modifications may be ascribed to both causation and reverse causation. Interestingly, we identified the major prognostic value of persistent liver dysfunction on continuation of anticancer treatment in patients with haematological malignancies discharged from ICU. Indeed, liver dysfunction may preclude the use of important cytotoxic agents such as anthracyclines, which are pivotal drugs in the treatment against acute leukemia and lymphoma, or methotrexate for the treatment of primary cerebral lymphoma or secondary neuro-meningeal invasion. Accordingly, 127 patients (74.7%) from the present study were scheduled to receive anthracyclines and/or methotrexate. In contrast, we did not identify any significant impact of persistent renal failure, as already suggested by another study [32]. Most patients were discharged with relatively low creatinine levels, unlikely to significantly impact on the intensity of cytotoxic chemotherapy. Moreover, most anticancer drugs used in first-line AML and NHL regimens (anthracyclines, low-dose cytarabine arabinoside, vincristine, corticosteroids) do not require dose adjustment with respect to the renal function.

Cancer treatment modifications dramatically impaired progression-free survival and overall survival at one year as compared to patients for whom the treatment could be continued as intended. Such major differences in outcomes can be ascribed to the fast and aggressive course of aggressive hematological malignancies, with treatment based on intensive cytotoxic chemotherapy. Similarly, other studies reported the negative impact of the use of delayed or reduced-dose chemotherapy regimens in patients with aggressive hematological malignancies [11–13]. The development of alternative therapeutic strategies with better tolerance, including targeted therapies or immunotherapy, may allow overcoming transient or definitive contra-indications to intensive cytotoxic chemotherapy.

The implications of our findings are relevant to both ICU physicians and haematologists. ICU admission policies of cancer patients have become much broader than it used to be while the related mortality steadily decreased. Nonetheless, it is of paramount importance to consider the expected benefits of critical care, not only in terms of ICU or hospital survival, but also in terms of long-term outcomes. Assessing a prognosis upon ICU admission is often challenging due to the unclear diagnosis of acute complications and the variability in treatment response, particularly concerning organ failures. The time of ICU discharge appears as a good point to anticipate the further therapeutic project, both in terms of hematological treatment and ICU readmission if needed. Indeed, early ICU readmissions may be required in the significant proportions of 19–34% of cancer patients who survived a first ICU stay, and have been associated with excess mortality [33, 34].

This work has several strengths and limitations. It focuses on a high-risk population with aggressive malignancies that require sustained intensive chemotherapy for several months. The single-center design allowed us a complete follow-up for almost all patients as well as an accurate collection of post-ICU management, although it may limit the external validation. We assume that our ICU admission policy based on close collaborations between intensivists and haematologists may result in a selected population. For instance, some patients considered at high risk of deterioration but no organ failure were admitted for close monitoring, and two thirds of them were actually able to continue their intended treatment [35]. However, ICU survivors from the present cohort and from the multicenter French and Belgian TRIAL-OH study, which comprised 59% of patients with AML or non-Hodgkin lymphoma, exhibited similar requirements for invasive mechanical ventilation (18%), vasopressors (21%) and renal replacement therapy (10%) [25]. Thanks to computerized patients’ data management systems, the number of missing quantitative data was low, but some important qualitative informations, including functional status and reasons for treatment modifications, were collected retrospectively from hand-written notifications. Finally, the study period ranged for 9 years, when ICU and hematology practices may have evolved but the limited number of patients precluded any temporal assessment. However, the treatments of AML and aggressive B-cell lymphoma rely on consistent guidelines for first-line and second-line treatment regimens, which did not significantly evolved over the study period.

## Conclusion

The intended cancer treatment could be resumed in a majority of ICU survivors with aggressive hematological malignancies. Mitigation or discontinuation of cancer treatment was associated with worsened one-year outcomes. At the time of ICU discharge, advanced age, persistent liver dysfunction and decisions to limit further life-support therapies were independent determinants of cancer treatment modifications. In the current era when most critically ill patients now survive the ICU stay, the overall therapeutic project should be reappraised in multidisciplinary discussion rounds at the time of ICU discharge.

## Electronic supplementary material

Below is the link to the electronic supplementary material.

Supplementary Material 1: table 1: Characteristics of patients with and without therapeutic limitations at ICU discharge. table 2: Characteristics of patients with inaugural acute myeloid leukemia admitted or not to the ICU within the first month following diagnosis. table 3: Characteristics of patients with B-cell Non-Hodgkin lymphoma admitted or not to the ICU within the first month following diagnosis.

## Data Availability

The datasets generated and/or analysed during the current study are not publicly available due to French regulations but are available from the corresponding author on reasonable request.

## Abbreviations

AML: Acute myeloid leukemia
B-NHL: B-non Hodgkin lymphoma
BL-IPI: Burkitt Lymphoma international prognostic index
CI: Confidence interval
HSCT: Hematopoietic stem cell transplantation
ICU: Intensive care unit
IPI: International prognostic index
IQR: Interquartile range
OR: Odds ratio
PS: Performans status
SAPS2: Simplified Acute Physiology Score 2
SOFA: Sequential Organ Failure Assessment
