# Supplementary material for: Impact of critical illness on continuation of anticancer treatment and prognosis of patients with aggressive hematological malignancies
Source: Ann Intensive Care. 2024 Sep 11;14:143. doi: 10.1186/s13613-024-01372-5 (PMC11390996; doi:10.1186/s13613-024-01372-5)
Supplement: Supplementary file 1 — Supplementary Material 1: table 1: Characteristics of patients with and without therapeutic limitations at ICU discharge. table 2: Characteristics of patients with inaugural acute myeloid leukemia admitted or not to the ICU within the first month following diagnosis. table 3: Characteristics of patients with B-cell Non-Hodgkin lymphoma admitted or not to the ICU within the first month following diagnosis. [file 13613_2024_1372_MOESM1_ESM.docx]

**Impact of critical illness on continuation of anticancer treatment and prognosis of patients with aggressive** **hematological malignancies**

Swann Bredin, Justine Decroocq, Clément Devautour, Julien Charpentier,

Clara Vigneron, Frédéric Pène

**Supplementary materials**

**Supplementary table 1** : Characteristics of patients with and without therapeutic limitations at ICU discharge

**Supplementary table 2**: Characteristics of patients with inaugural acute myeloid leukemia admitted or not to the ICU within the first month following diagnosis

**Supplementary table 3** : Characteristics of patients with B-cell Non-Hodgkin lymphoma admitted or not to the ICU within the first month following diagnosis

| Variables, n (%)  or median [IQR] | Therapeutic limitations  (n = 12) | No therapeutic limitations  (n= 158) | p |
| --- | --- | --- | --- |
| Age (years) | 74 [65-85] | 60 [46-72] | 0.002 |
| Male gender | 7 (58.3) | 87 (55.0) | 0.494 |
| Hematological malignancy |  |  | 0.035 |
| Acute myeloid leukemia | 10 (83.3) | 82 (51.8) |  |
| Non-Hodgkin lymphoma | 2 (16.7) | 76 (48.2) |  |
| Disease status |  |  | 0.129 |
| Inaugural | 7 (58.3) | 109 (68.9) |  |
| Relapse or progression | 4 (33.3) | 30 (18.9) |  |
| Reason for ICU admission |  |  | 0.009 |
| Acute respiratory failure | 7 (58. 3) | 26 (16.4) |  |
| Circulatory failure | 3 (25.0) | 46 (29.1) |  |
| Metabolic disturbances | 0 | 23 (14.5) |  |
| Neurologic disorder | 0 | 12 (7.5) |  |
| Septic shock | 2 (16.6) | 13 (8.2) |  |
| Monitoring | 0 | 37 (23.4) |  |
| Admission severity scores |  |  |  |
| SOFA score | 7 [4-10] | 5 [4-8] | 0.033 |
| SAPS2 | 46 [41-65] | 41 [30-51] | 0.043 |
| In-ICU management |  |  |  |
| Invasive mechanical ventilation | 4 (33.3) | 26 (16.4) | 0.153 |
| Vasopressors | 5 (41.6) | 24 (15.1) | 0.051 |
| Renal replacement therapy | 1 (8.3) | 15 (9.4) | 0.658 |
| Chemotherapy | 10 (83.3) | 95 (60.1) | 0.096 |
| In-ICU length of stay (days) | 4 [1-6] | 3 [2-6] | 0.537 |
| Characteristics at ICU discharge |  |  |  |
| Performans status (3-4) | 7 (58.3) | 29 (18.3) | 0.004 |
| Bilirubinemia > 20 µmol/L | 2 (16.6) | 27 (17.0) | 0.665 |
| Albuminemia < 25 g/L | 4 (33.3) | 41 (25.9) | 0.396 |
| Oxygen requirement | 7 (58.3) | 31 (19.6) | 0.006 |
| Acute kidney injury | 1 (8.3) | 32 (20.2) | 0.282 |
| Post-ICU outcomes |  |  |  |
| Hospital survival | 7 (58.3) | 140 (88.6) | 0.021 |
| 1-year survival | 3 (25.0) | 98 (62.0) | 0.001 |
| Remission | 1 (8.3) | 19 (12.0) |  |

**Supplementary table 1** : Characteristics of patients with and without therapeutic limitations at ICU discharge

| Variable N (%) or median [IQR] | Admitted in ICU  N = 73 | Not admitted in ICU  N = 104 | p |
| --- | --- | --- | --- |
| Demographics |  |  |  |
| Age (years) | 59 [44-71] | 63 [47-72] | 0.333 |
| Male gender | 37 (50.6) | 49 (47.1) | 0.640 |
|  |  |  |  |
| Cytogenetic risk classification |  |  | 0.891 |
| *Favorable* | 14 (19.1) | 23 (22.1) |  |
| *Intermediate* | 31 (42.4) | 42 (40.3) |  |
| *Poor* | 28 (38.3) | 39 (37.5) |  |
|  |  |  |  |
| Survival outcomes |  |  |  |
| 6-month overall survival | 51 (69.9) | 88 (84.6) | 0.019 |
| 1-year overall survival | 42 (57.5) | 81 (77.9) | 0.004 |

**Supplementary table 2 : Characteristics of patients with inaugural acute myeloid leukemia admitted or not to the ICU within the first month following diagnosis**

| Variable N (%) or median [IQR] | Admitted in ICU  N = 43 | Not admitted in ICU  N = 152 | p |
| --- | --- | --- | --- |
| Demographics |  |  |  |
| Age (years) | 62 [44-72] | 68 [53-73] | 0.239 |
| Male gender | 26 (60.4) | 77 (50.6) | 0.255 |
|  |  |  |  |
| Risk classification |  |  |  |
| Stage III/IV | 42 (97.6) | 129 (84.8) | 0.024 |
| IPI score >2 | 31 (72.0) | 75 (49.3) | 0.008 |
| Lymphoma subtype |  |  | 0.065 |
| *DLBCL* | 31 (72.0) | 132 (86.8) |  |
| *Burkitt lymphoma* | 9 (20.9) | 16 (10.5) |  |
| *Primitive cerebral lymphoma* | 3 (6.9) | 4 (2.6) |  |
|  |  |  |  |
| Survival outcomes |  |  |  |
| 6-month overall survival | 36 (83.7) | 135 (88.9) | 0.369 |
| 1-year overall survival | 30 (69.7) | 120 (78.9) | 0.207 |

**Supplementary table 3: Characteristics of patients with B-cell Non-Hodgkin lymphoma admitted or not to the ICU within the first month following diagnosis**

IPI : international prognostic index, DLBCL diffuse large B cell lymphoma
